# Supplementary material for: Chemical Composition, Antimicrobial and Antioxidant Bioautography Activity of Essential Oil from Leaves of Amazon Plant Clinopodium brownei (Sw.)
Source: Molecules. 2023 Feb 11;28(4):1741. doi: 10.3390/molecules28041741 (PMC9962765; doi:10.3390/molecules28041741)

Figure S1.

C. Brownei **Essential oil Chromatogram, in a Termo Scientific TR-5MS (5% -phenyl-95% dimethyl) polysiloxane) column.**

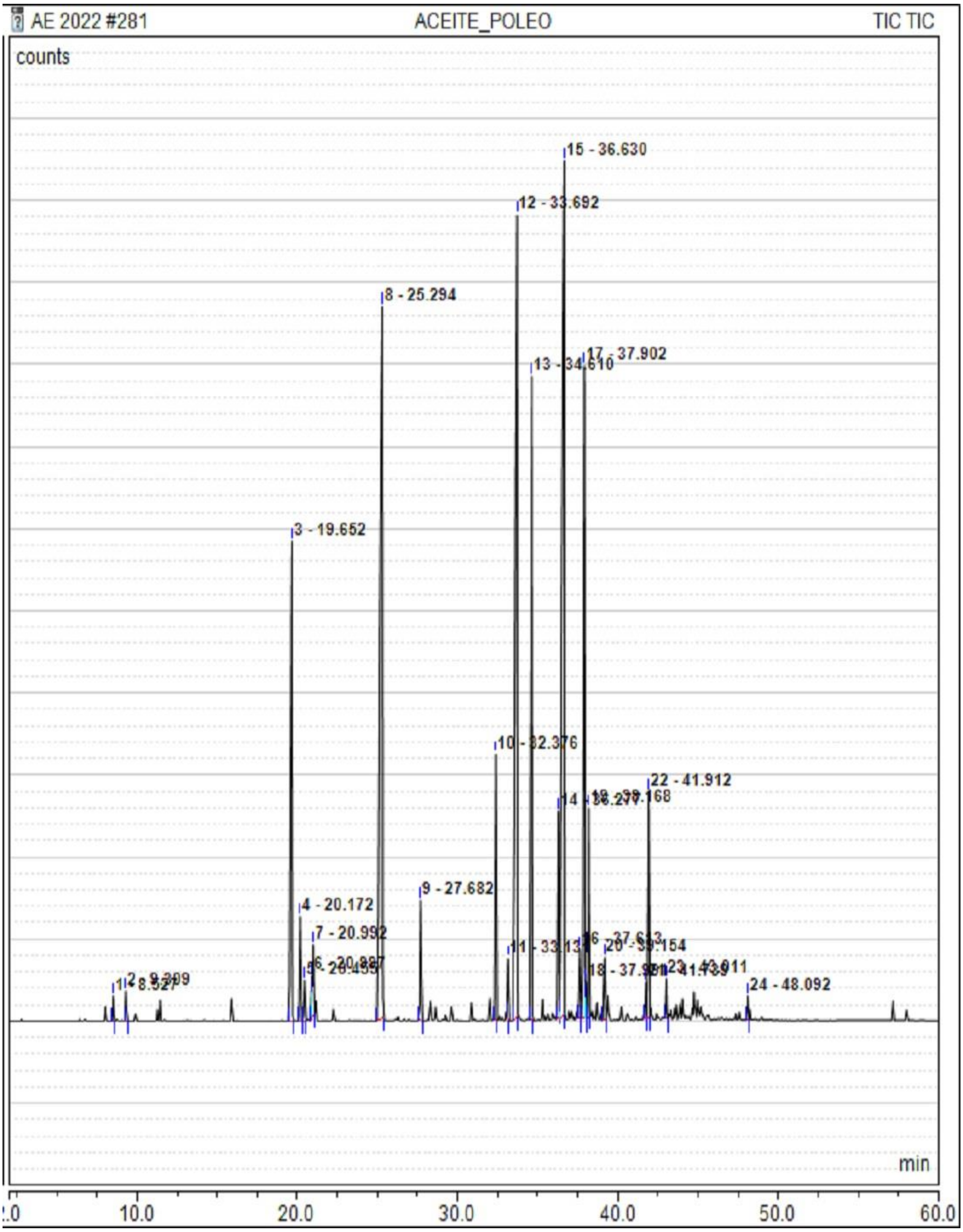

Figure S2.

***C. brownei* Essential Oil Chomatogram, in a Agilent DBWax (polyethylene glycol) columna.**

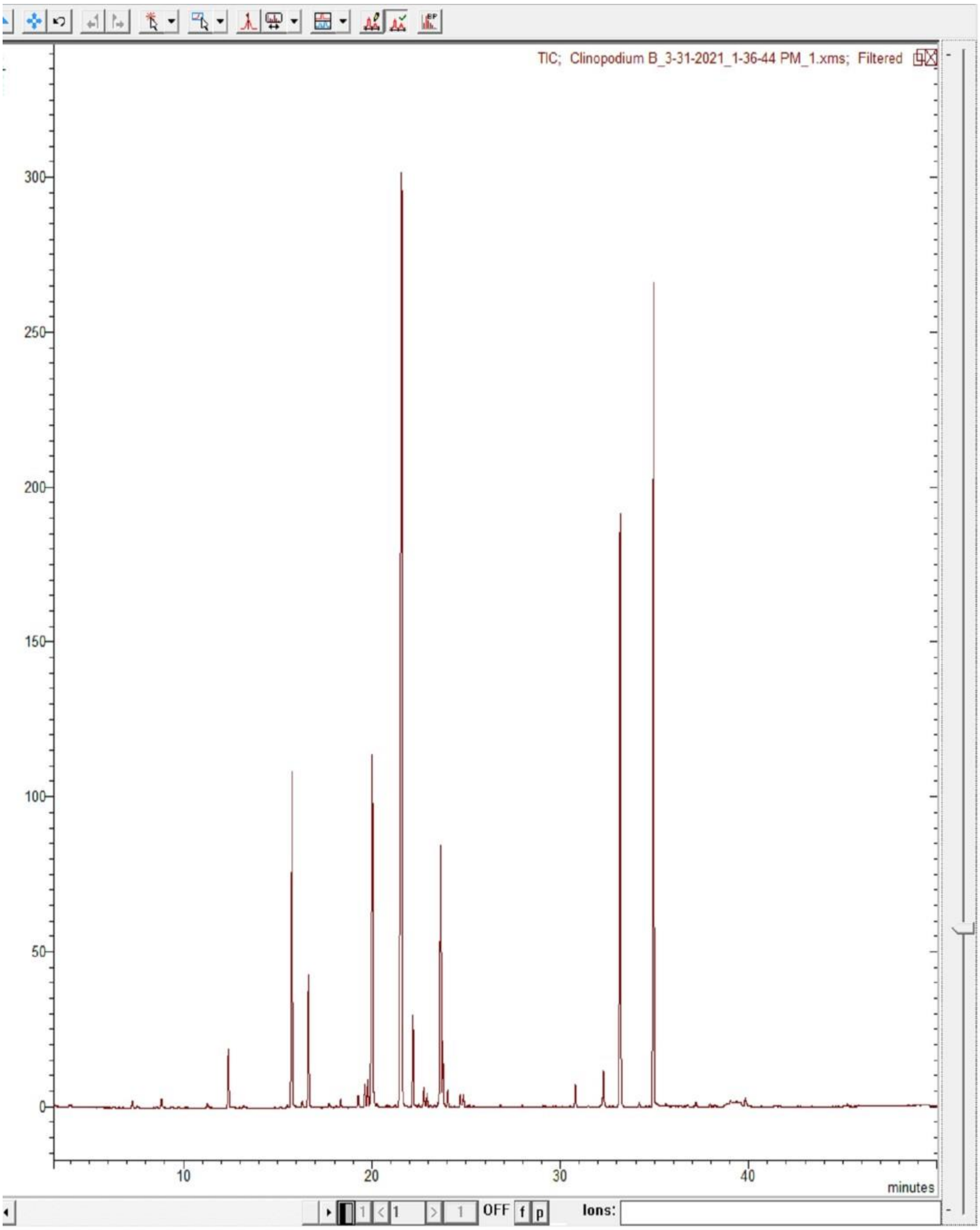

Figure S3.

Methyl cinnamate

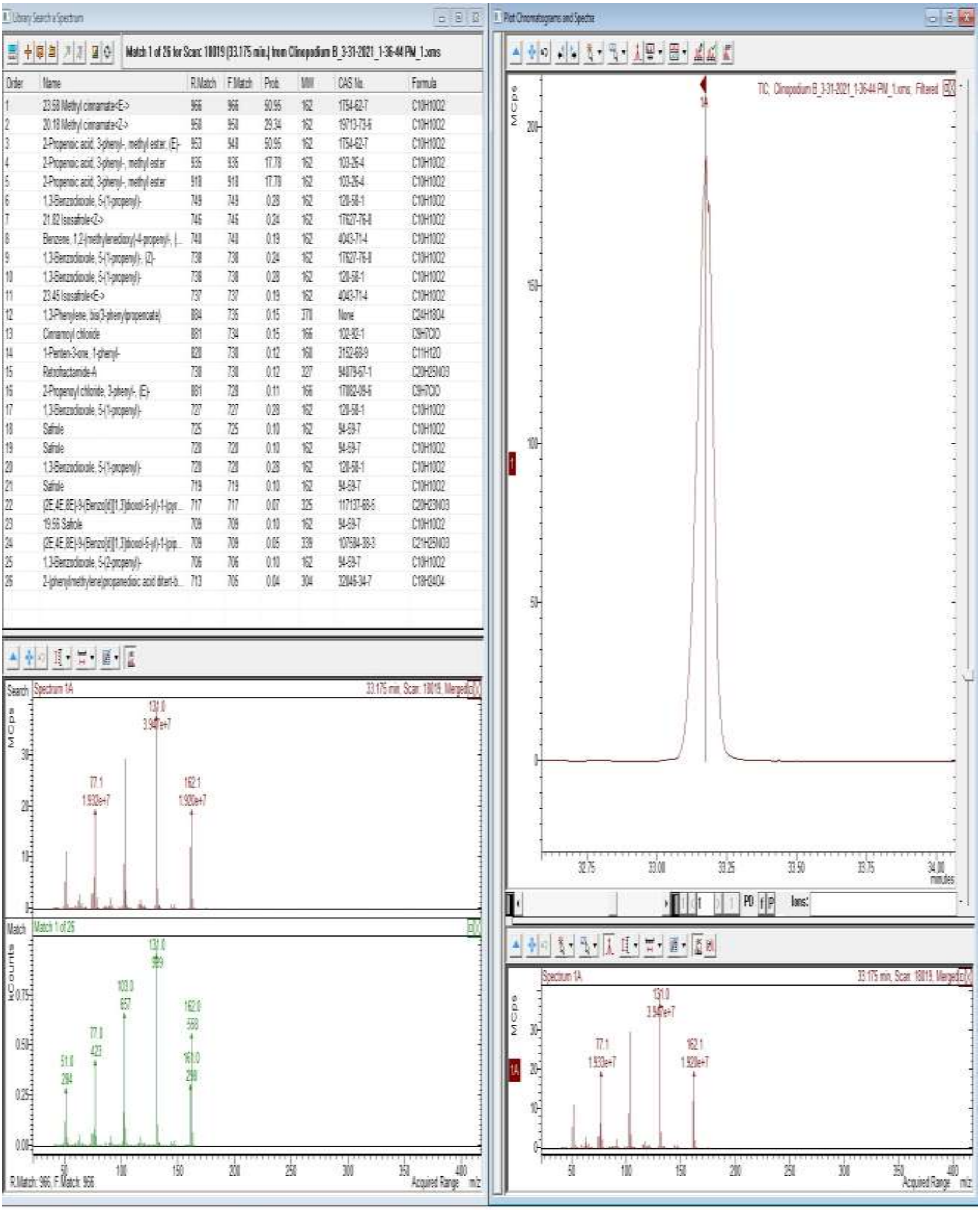

Figure S4

Ethyl cinnamate

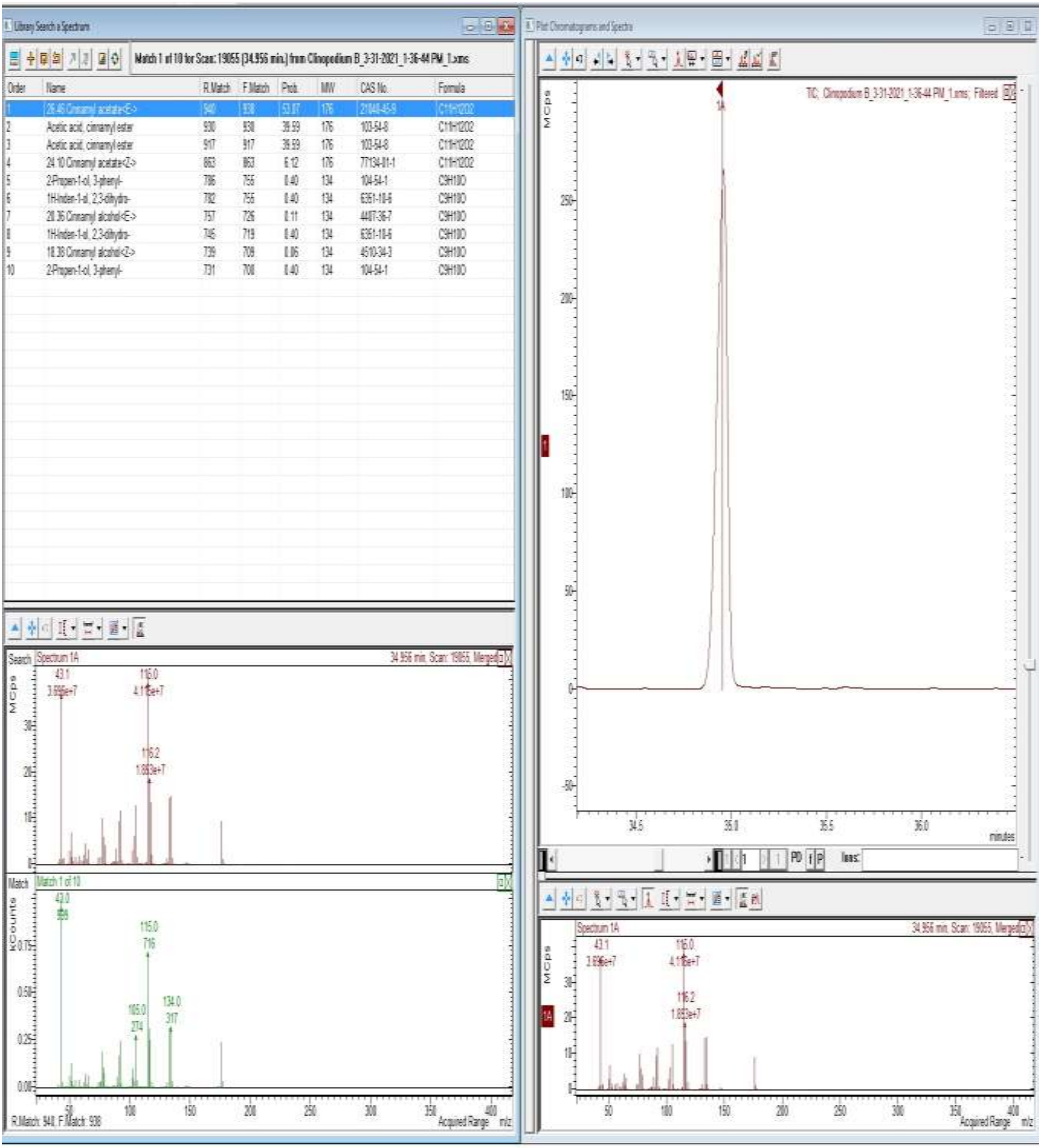

Figure S5.

Caryophyllene

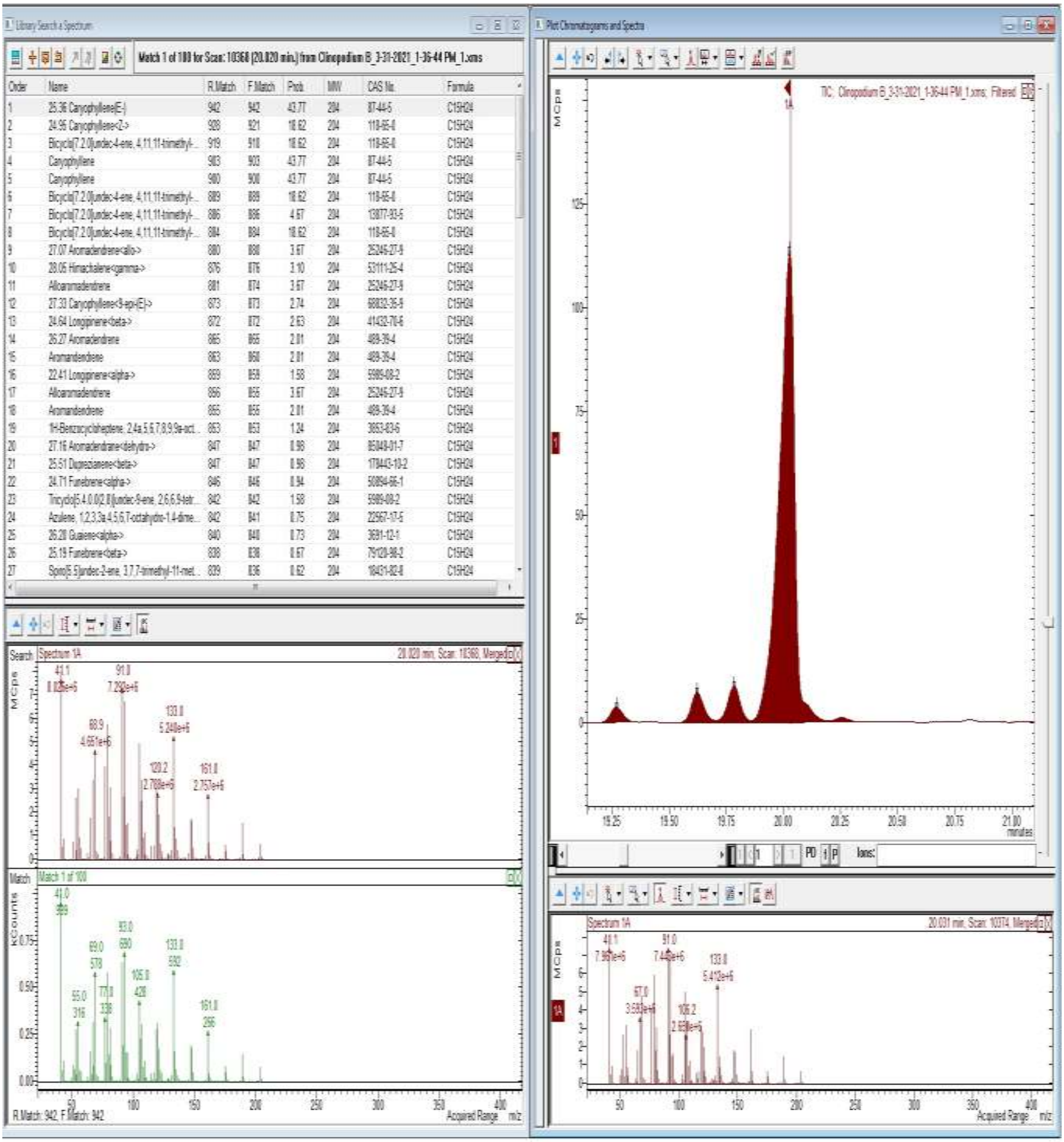

Supplement: Supplementary file 1 [file molecules-28-01741-s001.zip › molecules-2149615-supplementary.pdf]
